# Transposon-Associated Small RNAs Involved in Plant Defense in Poplar

**DOI:** 10.3390/plants14081265

**Published:** 2025-04-21

**Authors:** Cui Long, Yuxin Du, Yumeng Guan, Sijia Liu, Jianbo Xie

**Affiliations:** 1 National Engineering Research Center of Tree Breeding and Ecological Restoration, Beijing Forestry University, No. 35, Qinghua East Road, Beijing 100083, Chinajbxie@bjfu.edu.cn (J.X.); 2 Key Laboratory of Genetics and Breeding in Forest Trees and Ornamental Plants, Ministry of Education, College of Biological Sciences and Technology, Beijing Forestry University, No. 35, Qinghua East Road, Beijing 100083, China; 3 College of Biological Sciences and Technology, Beijing Forestry University, No. 35, Qinghua East Road, Beijing 100083, China; 4 Beijing Advanced Innovation Center for Tree Breeding by Molecular Design, Beijing Forestry University, No. 35, Qinghua East Road, Beijing 100083, China

**Keywords:** small RNAs, defense response, infection, poplar, *Melampsora larici-populina*

## Abstract

Utilizing high-throughput Illumina sequencing, we examined how small RNA (sRNA) profiles vary in Chinese white poplar (*Populus tomentosa*) across two pivotal infection stages by the rust fungus *Melampsora larici-populina*: the biotrophic growth phase (T02; 48 h post infection) and the urediniospore development and dispersal phase (T03; 168 h), both essential for plant colonization and prolonged biotrophic engagement. Far exceeding random expectations, siRNA clusters predominantly arose from transposon regions, with pseudogenes also contributing significantly, and infection-stage-specific variations were notably tied to these transposon-derived siRNAs. As the infection advanced, clusters of 24 nt siRNAs in transposon and intergenic regions exhibited pronounced abundance shifts. An analysis of targets indicated that *Populus* sRNAs potentially regulate 95% of *Melampsora larici-populina* genes, with pathogen effector genes showing heightened targeting by sRNAs during the biotrophic and urediniospore phases compared to controls, pointing to selective sRNA-target interactions. In contrast to conserved miRNAs across plant species, *Populus*-specific miRNAs displayed a markedly greater tendency to target *NB-LRR* genes. These observations collectively highlight the innovative roles of sRNAs in plant defense, their evolutionary roots, and their dynamic interplay with pathogen coevolution.

## 1. Introduction

Regulating gene expression, maintaining genome stability and chromatin structure, and influencing plant development like seed germination, small RNAs (sRNAs) serve as critical mediators in diverse biological processes [1,2,3,4]. Spanning roughly 21 to 24 nucleotides (nt), these sRNAs typically inhibit gene function, fitting within the broader RNA-silencing mechanism [5]. 

Two major classes of endogenous sRNAs have been identified in plants, namely, microRNAs (miRNAs) and small interfering RNAs (siRNAs) [5,6]. Plant miRNAs are transcribed from imperfect hairpin-shaped precursors and are typically 21 nt long, while siRNAs are 21–24 nt in length and are generated from double-stranded RNA duplexes [7]. miRNAs reduce the protein abundances of their targets through the direct cleavage or posttranscriptional regulation of target mRNAs to reduce their translation [8], which has important functions in a wide range of biological processes [5]. In *Arabidopsis*, over 100 miRNAs have been shown to be important for plant development [9] and abiotic stress tolerance [10].

siRNAs have been sequenced in many plant species, such as *Arabidopsis thaliana*, rice (*Oryza sativa*), and wheat (*Triticum aestivum*) [11,12,13,14], and have the ability to silence their targets by directly complementing their transcripts to prevent their translation. Studies in maize (*Zea mays*) have revealed that sRNA populations vary between parents and their progeny, contributing to the dramatic vigor of maize hybrids [15].

Strengthening plant resistance against pathogenic challenges, small RNAs (sRNAs) hold a crucial position, as evidenced by growing studies [16,17,18]. For instance, during *Arabidopsis* infection by *Pseudomonas syringae* or following exposure to bacterial flagellin, shifts in levels of specific miRNAs like miR393, miR160, and miR773 have been observed [19,20,21]. Through this innate immunity, a long-established protective mechanism, plants counteract a wide array of pathogens [17,18].

Despite the sequencing of thousands of endogenous siRNAs [22], their defensive roles in plants remain largely unexplored, necessitating deeper investigation. A superfamily of miRNAs, including miR482 and miR2118, engages with plant *NB-LRR* (nucleotide-binding leucine-rich-repeat) defense genes, as revealed by evolutionary analyses [23]. In rice and other monocots, miR2118 initiates phased secondary siRNAs (phasiRNAs) from noncoding RNAs, bolstering both pathogen resistance and male fertility [24], while, in tomato (*Solanum lycopersicum*), miR482 variants target the conserved P-loop motif to downregulate NLR transcripts, a suppression lifted by viral infections like cucumber mosaic virus to trigger resistance genes [25]. Contributing to antibacterial defenses, sRNAs also operate within eukaryotic host cells [26]. To uncover the functions of these vital genomic components, association genetics—characterizing polymorphisms and linking them to phenotypes—offers a promising approach.

Notably, understanding sRNA-mediated defense is particularly relevant for woody plants, which face unique challenges due to their distinct biological traits. Woody plants have distinct features that distinguish them from most herbaceous plants, including their large size, long lifespans, and perennial growth [27]. Plant growth and wood formation are complex and dynamic processes requiring the coordinated regulation of diverse metabolic pathways and trade-offs with immune responses [28,29]. It is almost certain that a perennial plant will encounter a pathogen before it can reproduce, and the long generation intervals of trees makes it difficult for them to match the evolutionary rates of microbes [30].

Encompassing over 6000 species, rust fungi (*Pucciniales*, *Basidiomycota*) pose significant threats to plants, with *Melampsora* spp. notably hindering bioenergy initiatives involving domesticated poplars due to insufficient lasting host resistance [31]. Fully sequenced genomes of the *Populus* genus make it an ideal system for studying sRNA functions in pathogen interactions. This genus includes key woody species such as western balsam poplar (*Populus trichocarpa*) [30], desert poplar (*Populus euphratica*) [32], and Chinese white poplar (*Populus tomentosa*) [33]. Certain *Populus* species stand out as preferred experimental models, benefiting from rapid growth and compact genomes.

Plants have the capacity to recognize pathogens through strategies involving both conserved and variable defense regulators, while pathogens suppress plant defense responses by delivering virulence effectors [34]. To gain deeper insights into the origins, evolutionary trajectories, and defensive impacts of poplar sRNAs, we sequenced the sRNAome of Chinese white poplar during the two essential stages of rust fungus (*Melampsora larici-populina*) infection: plant colonization and biotrophic growth. These analyses reveal the evolutionary importance and functional novelty of the sRNAs.

## 2. Materials and Methods

### 2.1. Plant Material and Fungal Treatments

Four-month-old Chinese white poplar (*Populus tomentosa*) plants were cultivated in pots with a soilless approach, housed in a climate-controlled glasshouse at 25 ± 1 °C and 50 ± 1% humidity, receiving 12 h of controlled natural light at 1250 μmol m^−2^ s^−1^ PAR. Infection studies employed *Melampsora larici-populina* strain G5, initiating infections by administering 5 μL of a 10^6^ conidia mL^−1^ suspension to six spots per leaf across three replicates per condition. Fully expanded leaves were detached from several poplar plants and spray-inoculated on their abaxial surface with 10^6^ urediniospores mL^−1^, with three biological replicates per treatment. Incubated at 25 ± 1 °C and 95% humidity in an LT36VL incubator (Percival Scientific, Inc., Perry, IA, USA), these leaves were sampled at 48 hpi (T02) and 168 hpi (T03). For controls (T01), leaves treated with sterile water were harvested at 0 hpi, and three independent biological replicates were performed. Samples were immediately frozen in liquid nitrogen and stored at −80 °C awaiting RNA analysis.

### 2.2. Library Preparation and NGS Sequencing

Using an RNeasy Plant Mini kit (Qiagen, Hilden, Germany) as per its instructions, total RNA was extracted, with on-column DNase treatment performed via RNase-Free DNase (Qiagen, Hilden, Germany) to remove DNA during the process. A Small RNA Sample Prep Kit (Illumina, Inc., San Diego, CA, USA) facilitated the attachment of 3′ and 5′ adapters to the RNAs, enabling those bearing both adapters to act as templates for cDNA generation through RT-PCR. Following purification, the cDNA was quantified using a Qubit dsDNA HS Assay Kit (Thermo Fisher Scientific, Waltham, MA, USA) on a Qubit 2.0 Fluorometer (Thermo Fisher Scientific, Waltham, MA, USA) and an Agilent 2100 Bioanalyzer (Agilent Technologies, Inc., Santa Clara, CA, USA), then subjected to clustering and sequencing on an Illumina HiSeq 2500 (Illumina, Inc., San Diego, CA, USA) according to the cBot and HiSeq 2500 protocols (Illumina, Inc., San Diego, CA, USA).

### 2.3. sRNAome and mRNA Analysis

To evaluate mature sRNA and mRNA transcript levels in leaves across three infection conditions, sequences were processed as follows: each library generated over 16 million raw reads, with 33.5–44.0% aligning to the *Populus trichocarpa* genome (version 3.0) for sRNAs and TopHat2 (version 2.1.1) [35] with parameters --*read-mismatches* 2 -*p* -G (permitting two mismatches) for mRNAs. The process of mapping was used Bowtie2 (version 2.4.5) [36]. These aligned sRNA reads were annotated by BLAST (version 2.11.0) against GenBank and Rfam (version 13; http://rfam.xfam.org/) with a one-mismatch threshold, while non-sRNA sequences—including tRNAs, rRNAs, snRNAs, snoRNAs, and scRNAs—were excluded. Unannotated sRNAs were then matched to miRBase 22.0 (http://www.mirbase.org/) with a tolerance of two mismatches, and novel miRNAs were predicted and identified using miRDeep2 (version 2.0.1). For mRNA analysis, transcript abundance and differential expression were determined via CuffDiff (version 2.2.1) with options -p -b [37].

To identify the siRNA clusters, the strategy used by [38] was applied, with some modifications. The 21–24-nt sRNAs from all the samples that did not match the miRNAs, ribosomal rRNA, or tRNAs and that could map to the *P. trichocarpa* genome were processed together. siRNAs within 100 bp of each other were merged into blocks referred to as siRNA clusters. The coordinates of the clusters were defined by the first and last siRNAs of the overlapping sequences. siRNA clusters with a transcription abundance of at least 5 tpm were included in the downstream analysis. The clusters were labeled according to the most abundant length of siRNA they contained (21, 22, or 24 nt), and the type of genomic region in which they were located.

### 2.4. Effector Prediction

Effector prediction was performed using a machine-learning program, EffectorP, with default parameters [39]. In this analysis, all of the 16,399 protein sequences in the *M. larici-populina* genome were used.

### 2.5. Target Prediction

The miRNA targets were annotated using the standard settings of psRNATarget [40] with an expectation value of 2.0 and top targets of 200. For targets in poplar, the transcripts of *P. trichocarpa* were downloaded from Phytozome 12 (https://phytozome.jgi.doe.gov/pz/portal.html, accessed on 18 April 2025). For targets in *M. larici-populina*, transcripts from this species were downloaded from the Ensemble database (ftp://ftp.ensemblgenomes.org/pub/release-39/fungi/fasta/melampsora_laricipopulina/cdna/).

### 2.6. Locations of miRNAs in the P. trichocarpa Genome

Within the *Populus trichocarpa* genome (Phytozome v12), siRNA clusters were mapped to genomic features—including transposons, introns, pseudogenes, intergenic regions, 5′ UTR, 3′ UTR, and 2 kb flanking zones of coding genes—with a minimum overlap of 80%, aiming to determine their overrepresentation via randomization tests. Genomic segments matching the length of these siRNA clusters were sampled randomly 1000 times, and their overlap with known elements was analyzed. A one-tailed *p*-value, reflecting the probability beyond the observed count, was derived from a Z-score calculated as the difference between the actual count and the mean of 1000 surrogate counts, normalized by the standard deviation of those surrogates, using a one-tailed Z test.

### 2.7. TE Annotation

Employing RepeatMasker (RM) version 4.0.6 with the Dfam_Consensus-20170127 and RepBase-20170127 databases tailored to *Populus* species, annotations for transposable elements (TEs) were generated. From these RM outputs, non-TE components—such as satellites, simple repeats, low-complexity sequences, and rRNAs—were filtered out.

### 2.8. Pseudogene Annotation

The intergenic sequences of the *P. trichocarpa* genome were used to identify the putative Pseudogenes. The overall pipeline used for this identification was generally based on the PlantPseudo workflow [41], and consisted of four major steps: (1) identify the masked intergenic regions with sequence similarity to known proteins using BLAST; (2) eliminate redundant and overlapping BLAST hits in places where a given chromosomal segment has multiple hits; (3) link homologous segments into contigs; and (4) realign sequences using tfasty to identify features that disrupt contiguous protein sequences.

## 3. Results

### 3.1. Poplar sRNAs Expressed During a Rust Fungus Infection

RNAs between 18 and 30 nt were extracted from Chinese white poplar under three conditions—mock-treated control (T01), biotrophic growth (T02), and urediniospore formation and release (T03)—and sequenced using an Illumina HiSeq 2500, generating 8.1 million clean reads (Table S1). These sRNA libraries enabled an examination of responses to *Melampsora larici-populina* infection across its key stages [42], with sequences processed to distinguish unique sRNAs for infected and control samples. Predominantly spanning 21 to 24 nt, typical of functional siRNAs and miRNAs, 56.6–65.4% of reads fell within this size range (Figure S1), and 33.4–44.0% aligned to the *Populus trichocarpa* genome v3.0 with up to one mismatch [30].

### 3.2. Pseudogenes and Transposons Act as Catalysts for the Formation of siRNA Clusters

**Figure 1:**
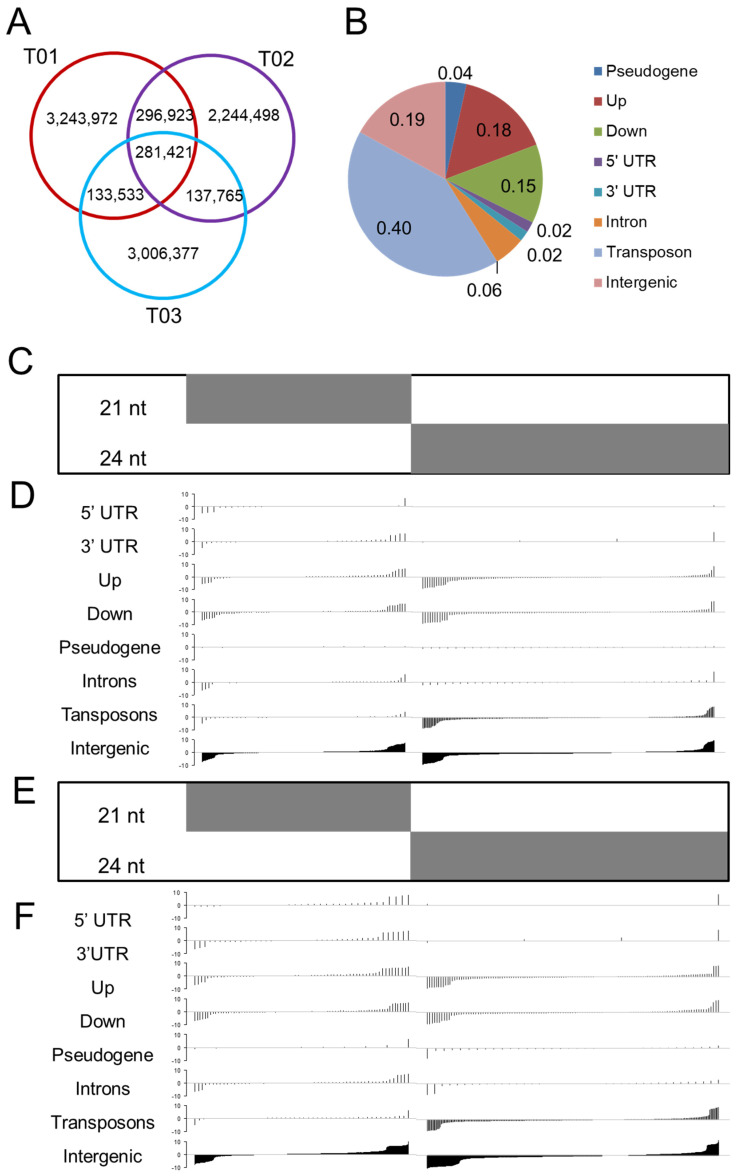
The stage differences in siRNA abundance primarily originate from transposons in response to infection. Shown are the 21 and 24 nt siRNA clusters with at least 5 tpm (transcripts per million mapped reads). Clusters are arranged in ascending order of fold change. (**A**) The common and stage-specific siRNA clusters. (**B**) The relative abundance of the siRNA clusters in the various types of physical locations in the *Populus* genome. (**C**–**F**) Degree of stage-specific differences for the siRNA clusters in the different locations. The log_10_ values of their abundance in T01 (control group; 48 h) divided by their abundance in (**C**,**D**) T02 (biotrophic growth phase; 48 h post infection) or (**E**,**F**) T03 (urediniospore formation and release phase; 168 h post infection). Up indicates the 2 kb upstream region of genes and down indicates the 2 kb downstream region of genes. The 22 nt and 24 nt siRNA clusters with at least 5 tpm in the three samples are shown. The clusters below the horizontal line were negatively regulated, while those above the horizontal line were positively regulated. The clusters are arranged in ascending order of fold change.

Pseudogene-derived siRNAs may co-opt transposon-targeting siRNA pathways, amplifying RNA interference (RNAi) signals to broadly silence homologous genomic regions, including transposon-rich loci, thereby linking genome plasticity to adaptive epigenetic regulation. We then investigated the genomic positions of siRNAs to uncover their evolutionary origins. The sRNA reads matching miRNA, tRNA, or ribosomal DNA sequences were removed from the datasets, and only those that mapped to the genome and were of the characteristic siRNA length (21–24 nt) were retained. The vast majority of siRNAs expressed during the rust fungus infection were found to be unique to one stage, while few siRNAs were common to all three samples (Figure 1A). Thus, infections could therefore create stage-specific siRNAs, producing a more complex and targeted siRNA population.

We used an approach similar to that reported by Barber et al. [15] to identify the types of genetic features associated with the stage-specific differences in siRNAs. We grouped overlapping 21–24-nt siRNAs within a ≤100-bp window on the *P. trichocarpa* genome into clusters based on their location within the genome. Totally, we identified 301,155 siRNA clusters. The relative abundances of the siRNA clusters were calculated (tpm: transcripts per million mapped reads). We then classified and annotated the siRNA clusters according to their common siRNA length (21, 22, or 24 nt) and their location in the genome. The 22 nt clusters accounted for less than 10% of all the clusters, and the 24 nt clusters accounted the largest proportion (>50%). The siRNA clusters with an abundance of at least 5 tpm in at least one stage were retained for the downstream analyses (Data S1).

Subsequently, we assessed where siRNAs reside within the *Populus* genome, spanning regions such as 5′ UTRs, 3′ UTRs, introns, 2-kb flanking sequences of coding genes, transposons, pseudogenes, and intergenic zones. Of the siRNA clusters, 625 (4%) were located within pseudogenes, 2973 (18%) were in upstream regions, 2586 (15%) were in downstream regions, 330 (2%) were in 5′ UTR regions, 395 (2%) were in 3′ UTR regions, 982 (6%) were in introns, 8159 (48%) were in transposons, and 4703 (19%) were in intergenic regions (Figure 1B).

The siRNA clusters were enriched for transposon element (TE)-associated sequences, as shown by the large percentages of siRNAs derived from the TE sequences (Figure 1B). Many of the TE sequences were annotated as high-copy retrotransposon families (Data S2); for example, 26.9% (2556) were long-terminal-repeat (LTR) sequences. Strikingly, siRNA clusters in pseudogenes and transposons occurred at rates far exceeding random expectation (*p* < 0.001, one-tailed z test; Table 1), indicating that these elements, particularly retrotransposons, likely fuel the emergence of certain *Populus* siRNAs.

### 3.3. Infection Stage-Specific Differences in the siRNAs Primarily Originate from the Transposon Regions

In the samples from the two infection stages, the proportions of 21 nt and 22 nt siRNAs included in any given cluster were significantly correlated (total r = 0.96; T01 r = 0.93; T02 r = 0.93; T03 r = 0.94; *p* < 2.2 × 10^−16^), and their proportions were also positively correlated with the 24 nt siRNAs (total r = 0.82 and r = 0.85 for 21 nt and 22 nt siRNAs, respectively; T01 r = 0.64 and r = 0.75, respectively; T02 r = 0.64 and r = 0.75, respectively; T03 r = 0.66 and r = 0.76, respectively; *p* < 2.2 × 10^−16^) (Data S1). The 21 nt clusters have a shorter mean length (205 bp) and were more abundant (80 tpm) than the 24 nt clusters (370 bp, 17 tpm; Wilcoxon rank-sum *p*-value < 2.2 × 10^−16^). To investigate the clusters exhibiting large stage-specific differences, we ordered the clusters by their absolute value of fold change values (T02/T01 or T03/T01). The degree of difference in the 24 nt siRNA cluster abundances between stages increased for those located in the transposon and intergenic regions (Figure 1C–F), with the strongest trend observed in the transposon regions.

**Figure 2:**
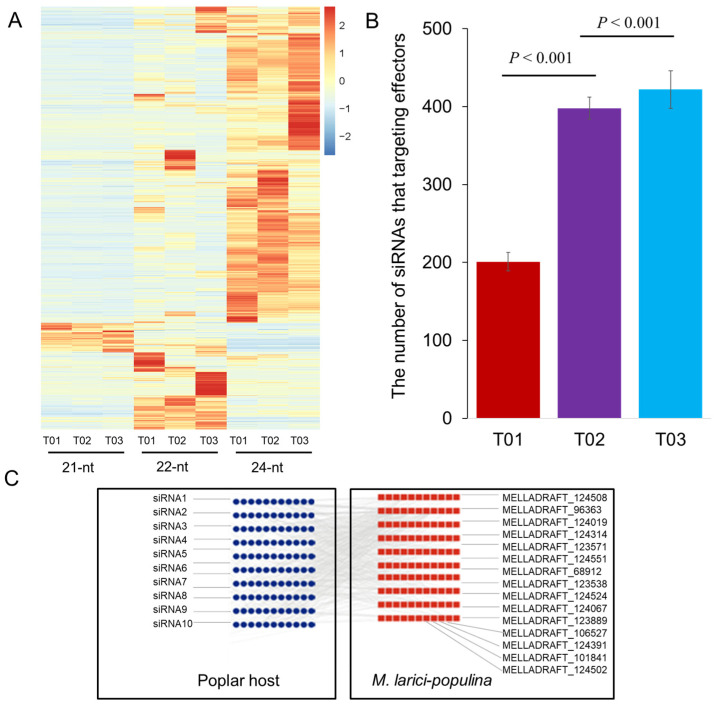
The difference in transposon siRNA activity are driven by 24 nt sRNAs. (**A**) The abundance of siRNAs in the three samples (T01; T02; and T03). The total abundance column displays the relative abundance of 21, 22, and 24 nt siRNAs. (**B**) The number of pathogen effectors targeted by the siRNAs in the three infection samples. The effectors were predicted by EffectorP and the target prediction were performed by using psRNATarget. T01: control group; 48 h. T02: biotrophic growth phase; 48 h post infection. T03: urediniospore formation and release phase; 168 h post infection. (**C**) A schematic model illustrating the role of poplar-derived siRNAs in regulating pathogenicity genes of *Melampsora larici-populina*. Error bars, bootstrap-based 95% confidence intervals on the mean estimates. Statistical inference is conducted with a permutation test on the mean (perm.test in the R package exact RankTests).

To further investigate the global differences in siRNA abundance in the retrotransposon families between the different infection stages, we mapped the 21 nt, 22 nt, and 24 nt siRNAs that perfectly matched the *Populus* genomes onto the retrotransposons. Figure 2A shows the retrotransposon families that showed an siRNA abundance of at least 100 tpm in at least one of the stages. The production of 21 nt siRNA lengths and the abundance of these siRNAs located in the retrotransposon families are more similar across the three stages than those of the 22 nt and 24 nt siRNAs.

The stage-specific differences in the abundance of siRNAs located in the transposons were examined using an X^2^ test for each siRNA length. The differences in the siRNA abundance for most transposon families between the stages (T02 vs. T01; and T03 vs. T01) was contingent on the 24 nt length sequences (*p* < 0.05; Chi-square test; Data S3; Figure 2A). The stage-specific differences in siRNA abundance for these retrotransposon families thus primarily resulted from the 24 nt siRNAs.

### 3.4. Populus siRNAs Mediate Plant–Pathogen Interactions, as Revealed Using a Shotgun Strategy

To explore the potential role of the *Populus* siRNAs in plant–pathogen interactions, we profiled the siRNA sequences that mapped to the *P. trichocarpa* genome which could regulate the genes of the fungal pathogen *Melampsora larici-populina*. In the biotrophic growth (T02) and urediniospore formation and release (T03) stages, more than 95.0% of the fungal genes (T02: 15,954; T03: 15,472; and total fungal genes: 16,372) were predicted to be targeted by siRNA sequences (Data S4). Nearly one-third of the expression of *Melampsora larici-populina* genes detected in our transcriptome were altered at least by twofold between sample pairs (T02/T01; T03/T01; and T02/T01) (Data S5). We hypothesize that some siRNAs could suppress pathogen pathogenicity by targeting pathogen virulence genes using a shotgun strategy. Importantly, if siRNA–pathogen interactions do not occur randomly, we would expect sRNA to preferentially target specific pathogen genes. As expected, compared with the control sample (T01), pathogen effectors were targeted by more siRNAs at the biotrophic growth and urediniospore formation and release phases (*p* < 0.001; permutation test; Figure 2B), suggesting there is a clear selection for the siRNA–target interactions.

### 3.5. The Populus-Specific miRNAs Are More Involved in the Regulation of the Disease-Resistance (DR) Genes

To investigate miRNAs in our sequencing data, recognized for their critical role in plant immunity [26,43], we mapped sRNA reads against Rfam (version 13) to annotate the sRNAome. Following the exclusion of scRNAs, tRNAs, rRNAs, snRNAs, and snoRNAs, the remaining 423 sRNA precursors were classified into 211 miRNA families, averaging 2.0 genes per family across the three infection stages. We found that 67.7% of the miRNAs could be annotated in miRBase (version 22); however, most of the putative novel miRNAs did not remain in the final dataset. These excluded miRNA sequences may not represent bona fide miRNAs or may be expressed at levels too low to reliably show that they have the characteristics of miRNAs (at least one read matching their star sequence; Data S6). The expression values of each miRNA gene were also calculated and normalized (Data S7).

**Figure 3:**
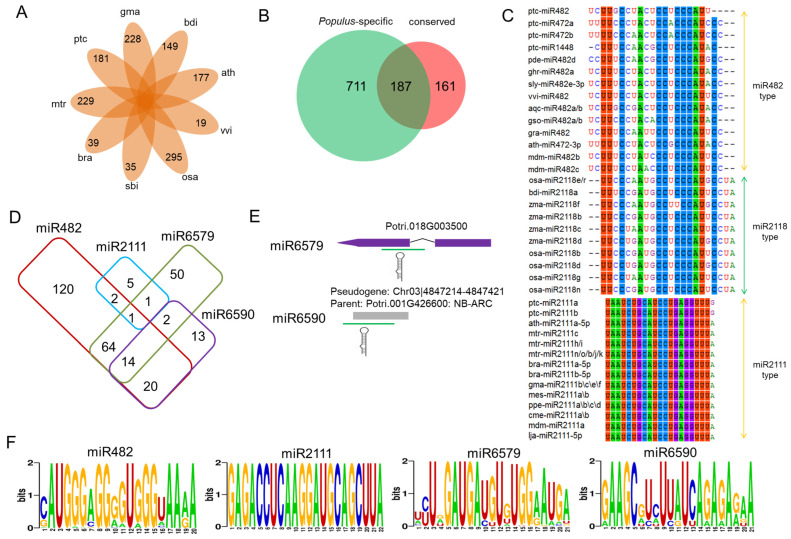
The *Populus NB-LRR* genes are targeted by *Populus*-specific miRNAs. (**A**) The species-specific miRNAs of nine plant species. ptc: *Populus trichocarpa*; mtr: *Medicago truncatula*; bra: *Brassica rapa*; sbi: *Sorghum bicolor*; osa: *Oryza sativa*; vvi: *Vitis vinifera*; ath: *Arabidopsis thaliana*; bdi: *Brachypodium distachyon*; gma: *Glycine max*. (**B**) Comparison of the number of predicted disease-resistance target genes of the conserved and *Populus*-specific miRNAs. (**C**) Conservation profile of miR482, miR2118, and miR2111 in diverse plant species. The mature miRNA sequences were retrieved from miRBase. (**D**) Comparison of the number of predicted *NB-LRR* genes targeted by miR482, miR2111, miR6579, and miR6590. (**E**) The genome locations of two *Populus*-specific miRNAs. (**F**) The consensus coding sequences of the targets of four *Populus* miRNAs.

Combining miRNAs identified here with those from closely related species in miRBase (v22), we distinguished *Populus*-specific miRNAs, finding 181 of 211 families unique to *Populus*, of which 70 aligned with prior findings (Figure 3A). Employing psRNATarget [40], we predicted 65,777 miRNA–target gene pairs with an expectation score of 5 (Data S8), and a subsequent expression analysis uncovered 1940 pairs exhibiting negative correlations (*p* < 0.05; Pearson correlation; Data S9). A public degradome dataset [44]; Data S5, available at https://nph.onlinelibrary.wiley.com/action/downloadSupplement?doi=10.1111%2Fnph.14046&file=nph14046-sup-0002-NotesS1-S12.xlsx, accessed on 18 April 2025) validated 553 pairs involving 165 miRNAs (Data S10), encompassing conserved pairs such as miR156-SPL, miR172-AP2, and miR397-LAC [45,46,47]. Excluding predicted disease-resistance (DR) gene targets from both conserved and *Populus*-specific sets left 161 DR targets for conserved miRNAs and 711 for *Populus*-specific ones, with 66% (120 of 181) of *Populus*-specific miRNAs regulating DR genes—far exceeding the 23.3% of conserved miRNAs (*p* = 0.009; Fisher’s exact test; Table 2, Data S11; Figure 3B).

To gain a better understanding of the functional roles of the *Populus*-specific miRNA families, we performed a pfam domain analysis of their predicted targets. The top 20 domains of the *Populus*-specific miRNA targets were highly correlated with the domains of the plant DR genes (Figure S2); for example, NB-ARC and LRR domains, which are known to dissociate upon effector-dependent activation [48], were overrepresented in the samples.

### 3.6. Populus-Specific miR6579 and miR6590 Target Numerous NB-LRR Genes

Plant NB-LRRs (encoded by *NB-LRR* genes) are commonly targeted by miRNAs, typically generating phased small interfering RNAs (phasiRNAs), which can reduce the levels of these targets in a cis or in a trans manner [44]. In the two stages of the rust fungus infection, a conserved miRNA superfamily composed of miR482 targeted the *NB-LRR*s at the encoded and conserved P-loop motifs. This family was found to be highly conserved among divergent plant species, including rice, *Arabidopsis*, grape (*Vitis vinifera*), and soybean (*Glycine max*) [23,49,50]. A second conserved family, miR2111, was identified in the eudicotyledons (Figure 3C), indicating the existence of this miRNA family prior to the divergence of these species from a common ancestor around 103 Mya [51]. The highly conserved 20 nt miR482s were predicted to target 221 *NB-LRR*s. Two related *Populus*-specific families, miR6579 and miR6590, which both contain 21 nt miRNAs, were predicted to target 132 and 49 NB-LRRs, respectively (Figure 3D–F). These two *Populus*-specific miRNA families had the highest transcript abundance during the mock-inoculated control plants, decreasing to a low level during the biotrophic growth (T02) and urediniospore formation and release (T03) stages (Data S7). By contrast, the highly conserved 22 nt miR2111s were predicted to target only nine *NB-LRR*s, three of which were also predicted targets of miR482, while two were predicted to be targeted by miR6579 (Figure 3D–F). These results indicate that the *NB-LRR*s in *Populus* are primarily targeted by conserved miR482s and *Populus*-specific miR6579s.

A careful examination of their precursor sequences revealed that miR6579 and miR6590 were intron-derived and pseudogene-derived miRNAs, respectively (Figure 3E). The parent of the pseudogene Chr03|4847214-4847421 was an *NB-LRR* gene (Potri.001G426600), suggesting that pseudogenes could act as catalysts for the formation of miRNAs. In poplar, the 21 nt miR6590 family showed characteristics canonical of the typical miRNA triggers associated with ARGONAUTE1 (AGO1) for the initiation of the PHAS loci, such as “U” in the 5′ position, “A” in position 10, and “C” in the 3′ position, indicating that this miRNA may trigger the biogenesis of secondary siRNAs [52]. It seems that species-specific miRNAs could become fixed in the regulatory network once a number of targets have evolved for which the presence of a miRNA-binding site would be beneficial.

## 4. Discussion

### 4.1. Transposons and Posttranscriptional Regulation

Assessing poplar siRNA distribution across genomic elements—including 5′ UTRs, 3′ UTRs, introns, 2-kb coding gene flanks, transposons, pseudogenes, and intergenic regions—we observed that transposons contributed 48% of siRNAs, with clusters notably tied to high-copy retrotransposon families. Long regarded as mere genomic noise, these ubiquitous transposons are now valued for their profound impact on eukaryotic genome structure, diversity, and flexibility [53], evolving faster than coding sequences due to greater mutation susceptibility [54]. Exhibiting distinct stage-specific fluctuations, the 24 nt siRNAs derived from these retrotransposons likely mediate posttranscriptional silencing by inducing repressive histone modifications, thus enhancing TE repression [55,56].

Our findings further illustrate that it is difficult to assess the connection between siRNAs and transposons in the process of plant defense. In general, the mobilization of transposons can be mutagenic [57], so host genomes have evolved elaborate mechanisms to suppress their activities. Most of the 24 nt siRNAs are likely to be involved in the transcriptional regulation of transposons through RNA-directed DNA methylation [1], but many TE-associated siRNAs were also found to influence non-TE transcripts exerting certain regulatory roles [58]; for example, 24 nt siRNAs were found to target pathogen genes [57]. Transposons are rapidly activated by biotic and abiotic stresses, however [59,60,61], potentially complicating the regulatory loop between the siRNAs and TEs.

siRNAs are an ideal tool for use in plant defense because of their target specificity and highly efficient responses [62,63] proposed that the retrotransposon-associated siRNAs may target genes with homologous sequences. Moreover, direct evidence in *Arabidopsis* suggests that 21 nt siRNAs derived from an Athila retrotransposon could posttranscriptionally regulate the Upstream binding protein 1b (*UBP1b*) gene to mediate the stress response [64]. In rice, transposon-derived 24 nt siRNAs could target the genes involved in hormone homeostasis, suggesting that transposon-associated regulatory modules could play a variety of roles in plant development and defense [65]. It is, therefore, possible that the 21–24 nt siRNAs may have another purpose besides the maintenance of genome stability, and may enhance plant defense by targeting the pathogen genes. Considering the high genetic diversity and mobilization of transposons, the regulatory system mediated by the TE-derived siRNAs could play a major role in the performance of plant defense.

### 4.2. Cross-Kingdom RNAi and sRNAs Mediate Plant–Pathogen Interactions

An endless arms race drives host–pathogen coevolution [66]. In plants, pathogens can invoke multiple layers of immune responses, and many defense genes were found to play key roles in this process [67,68]. Most potential pathogens are blocked by nonhost resistance and pathogen-associated molecular pattern (PAMP)-triggered immunity (PTI) [62]; however, many pathogens deliver a variety of effectors into plant cells to suppress host immunity. To counteract this attack, plants have evolved resistance proteins and immune mechanisms to sense these effectors and trigger a robust resistance response [69]. Plant sRNAs are essential regulatory noncoding RNAs that can posttranscriptionally silence their target genes [62,70]. Recent progress in profiling sRNAs, especially advances in next-generation sequencing technology, has revealed their extensive and complicated involvement in interactions between plants and viruses, bacteria, and fungi [70].

Fungal pathogens deliver sRNAs into plant cells to induce cross-kingdom RNAi of plant immunity genes, a mechanism initially identified by Weiberg et al. [43] and further elucidated through extracellular vesicle-mediated transport [71,72]. A similar strategy was also observed in animal–parasite systems [73]. Furthermore, sRNAs generated from the host plant cells can also be transferred into pathogen cells [66], as fungal pathogens are known to be capable of taking up external sRNAs and long dsRNAs [66,74]. Pathogen genomes encode variable effector repertoires for suppressing host immunity [75]; specifically, these effectors are often encoded in genomic regions of high plasticity [76], such as TEs [77]. Given that effectors are often clustered in these regions, TEs must function as an evolutionary force shaping these virulence factors by contributing to their diversification [77]. Sequence diversification is particularly important for pathogen effectors, which are essential components facilitating their coevolution with, and outmaneuvering of, the host.

Considering the high diversification of virulence factors generated by pathogens, it is reasonable to assume that transposon-derived sRNAs are also the best sources of new regulators for plant defense resistance, facilitating cross-kingdom RNAi. TEs, the most abundant component of most eukaryote genomes, are hotspots for generating sRNAs, which is particularly true of the retrotransposons. Here, we found that pathogen effectors were targeted by more poplar siRNAs in the biotrophic growth and urediniospore formation and release stages of infection by a rust fungus, in comparison with control. It is, therefore, reasonable to conclude that the activation of the siRNA repertoire does not occur randomly, and that their targets are instead involved in more central roles in the plant–pathogen interactions. Supporting this hypothesis, a previous study reported that the transfer of host sRNAs could silence virulence-related genes and suppress fungal pathogenicity in *Arabidopsis* [71].

From a plant breeding perspective, the siRNA-mediated cross-kingdom RNAi mechanism offers a promising strategy for engineering durable disease resistance. For instance, selecting or editing crop varieties to amplify siRNA production targeting conserved pathogen effectors could provide broad-spectrum resistance while minimizing evolutionary constraints imposed by single-gene R strategies [78]. Furthermore, the dynamic interplay between transposon-derived siRNAs and rapidly evolving pathogen virulence factors suggests that leveraging TE-rich genomic regions as “sRNA reservoirs” may enhance adaptive immunity in perennial crops like poplar. This approach aligns with evolutionary breeding principles, where harnessing natural genomic plasticity could yield cultivars capable of counteracting pathogen coevolution [79]. Critically, integrating sRNA profiling into marker-assisted selection programs may accelerate the identification of elite germplasm with enhanced RNAi-based defense traits, particularly against rust fungi exhibiting high effector diversification rates.

### 4.3. Disease Resistance Associated with Populus-Specific miRNAs

Acting as key detectors in effector-triggered immunity, plant *NB-LRR* genes—categorized into TIR-NB-LRRs with a Toll/Interleukin-1 receptor-like domain and CC-NB-LRRs with a coiled-coil domain [80]—fortify innate defenses against a wide range of pathogens and pests [81], with miRNAs triggering phasiRNA production to reduce transcript abundance locally and across genes [82]. These genes, marked by frequent duplication, loss, structural changes, subfamily diversification, and species-specific copy number variations [81,83,84], contrast with stable-copy genes, driving rapid, lineage-specific immune adaptations. While conserved miRNAs often target conserved domains like P-loop motifs across families [52,85], newer miRNAs exhibit less specificity, potentially binding mRNAs less selectively [86]. In *Populus*, the salicoid whole-genome duplication (WGD) event, common to Salicaceae, has enriched genes in specialized functional groups [87], with targets of recently emerged miRNAs spanning DR *NB-LRR* genes, ABC transporters, and select transcription factors, aligning with retained duplicates in this genus [30]. Our analysis shows *Populus*-specific miRNAs, despite fewer total targets, surpass conserved miRNAs in regulating *NB-LRR* genes, reflecting a defense-linked diversity that likely governs newly evolved DR genes, boosting host adaptability. Over time, these emerging miRNAs may refine their roles within regulatory networks, extending control to additional specialized targets [44,86], shedding light on siRNA’s origins and regulation in *Populus trichocarpa* against *Melampsora larici-populina*.

## Figures and Tables

**Table 1:** Enrichment of siRNA clusters in various regions of *Populus trichocarpa* genome.

Position	^a^ Expected (SD)	Observed	Z Score ^b^	*p*-Value
Pseudogene	475.80 (6.16)	625	24.22	1.39 × 10^−127^
Up	3192.40 (23.28)	2973	−9.42	2.26 × 10^−21^
Down	3078.80 (33.52)	2586	−14.7	3.22 × 10^−49^
5′ UTR	450.60 (20.88)	330	−5.78	3.74 × 10^−9^
3′ UTR	570.60 (9.28)	395	−18.92	3.90 × 10^−80^
Intron	2923.40 (27.52)	982	−70.55	0.00
Transposon	4271.00 (39.6)	8159	98.18	0.00
Intergenic	4731.40 (59.68)	4703	−0.47	0.32

^a^ 1000 randomly generated siRNA clusters datasets in the genome background. ^b^ Significant deviation from expected proportions (one-tailed z test). Note that some siRNA clusters were within two types of genomic regions.

**Table 2:** Comparison of the targets of *Populus*-specific and conserved miRNAs.

No. of Conserved miRNA ^a^	No. of *Populus*-miRNAs	Conserved miRNAs Targeting Disease Resistance Genes ^b^	*Populus*-Specific miRNA Targeting Disease Resistance Genes ^c^	Adjusted *p*-Value ^d^
30	181	7	120	0.009

^a^ Number of conserved miRNA genes that have targets. ^b^ Number of conserved miRNAs targeting disease resistance genes. ^c^ Number of *Populus*-specific miRNA targeting disease resistance genes. ^d^ BH-adjusted *p*-value was derived from Fisher’s exact test.

## Data Availability

Raw data of the RNA sequencing and small RNA sequencing are available for download at the BIGD Genome Sequence Archive under accession number CRA001965.

## Supplementary Materials

The following supporting information can be downloaded at: https://www.mdpi.com/article/10.3390/plants14081265/s1, Figure S1. Length distribution of small RNA reads. Figure S2. The top 20 Pfam domains of *Populus*-specific miRNA targets. Figure S3. The procedure used for the library construction. Table S1. Small-RNA-sequencing reads produced for the three samples. Data S1. siRNA clusters identified in the three samples of rust infection. Data S2. The siRNA clusters in transposons. Data S3. The differences in the siRNA abundance for transposon families between the stages. Data S4. The *Melampsora larici-populina* targets of plant siRNAs. Data S5. The *Melampsora larici-populina* genes expressed during the infection of *Populus tomentosa*. Data S6. The predicted novel miRNAs. Data S7. The expression value of each miRNA. Data S8. The predicted targets of the miRNAs. Data S9. Correlation of the miRNA/target pairs. Data S10. The miRNA/target pairs with degradome evidence. Data S11. The miRNA/target pairs predicted using psRNATarget.

## References

[1] Matzke M.A., Birchler J.A. (2005). RNAi-mediated pathways in the nucleus. Nat. Rev. Genet..

[2] Ghildiyal M., Zamore P.D.J.N.R.G. (2009). Small silencing RNAs: An expanding universe. Nat. Rev. Genet..

[3] Liu P.P., Montgomery T.A., Fahlgren N., Kasschau K.D., Nonogaki H., Carrington J.C. (2007). Repression of AUXIN RESPONSE FACTOR10 by microRNA160 is critical for seed germination and post-germination stages. Plant J..

[4] Olovnikov I., Aravin A.A., Toth K.F. (2012). Small RNA in the nucleus: The RNA-chromatin ping-pong. Curr. Opin. Genet. Dev..

[5] Carthew R.W., Sontheimer E.J. (2009). Origins and Mechanisms of miRNAs and siRNAs. Cell.

[6] Nykänen A., Haley B., Zamore P.D. (2001). ATP requirements and small interfering RNA structure in the RNA interference pathway. Cell.

[7] Harding J.L., Horswell S., Heliot C., Armisen J., Zimmerman L.B., Luscombe N.M., Miska E.A., Hill C.S. (2014). Small RNA profiling of Xenopus embryos reveals novel miRNAs and a new class of small RNAs derived from intronic transposable elements. Genome Res..

[8] Khraiwesh B., Arif M.A., Seumel G.I., Ossowski S., Weigel D., Reski R., Frank W. (2010). Transcriptional control of gene expression by microRNAs. Cell.

[9] Mallory A.C., Vaucheret H. (2006). Functions of microRNAs and related small RNAs in plants. Nat. Genet..

[10] Sunkar R., Zhu J.K. (2004). Novel and stress-regulated microRNAs and other small RNAs from Arabidopsis. Plant Cell.

[11] Ha M., Lu J., Tian L., Ramachandran V., Kasschau K.D., Chapman E.J., Carrington J.C., Chen X., Wang X.-J., Chen Z.J. (2009). Small RNAs serve as a genetic buffer against genomic shock in Arabidopsis interspecific hybrids and allopolyploids. Proc. Natl. Acad. Sci. USA.

[12] Hamilton A.J., Baulcombe D.C. (1999). A species of small antisense RNA in posttranscriptional gene silencing in plants. Science.

[13] He G., Zhu X., Elling A.A., Chen L., Wang X., Guo L., Liang M., He H., Zhang H., Chen F. (2010). Global epigenetic and transcriptional trends among two rice subspecies and their reciprocal hybrids. Plant Cell.

[14] Kenan-Eichler M., Leshkowitz D., Tal L., Noor E., Melamed-Bessudo C., Feldman M., Levy A.A.J.G. (2011). Wheat hybridization and polyploidization results in deregulation of small RNAs. Genetics.

[15] Barber W.T., Zhang W., Win H., Varala K.K., Dorweiler J.E., Hudson M.E., Moose S.P. (2012). Repeat associated small RNAs vary among parents and following hybridization in maize. Proc. Natl. Acad. Sci. USA.

[16] Pumplin N., Voinnet O. (2013). RNA silencing suppression by plant pathogens: Defence, counter-defence and counter-counter-defence. Nat. Rev. Microbiol..

[17] Peláez P., Sanchez F. (2013). Small RNAs in plant defense responses during viral and bacterial interactions: Similarities and differences. Front. Plant Sci..

[18] Wong J., Gao L., Yang Y., Zhai J., Arikit S., Yu Y., Duan S., Chan V., Xiong Q., Yan J. (2014). Roles of small RNAs in soybean defense against Phytophthora sojae infection. Plant J..

[19] Navarro L., Dunoyer P., Jay F., Arnold B., Dharmasiri N., Estelle M., Voinnet O., Jones J.D. (2006). A plant miRNA contributes to antibacterial resistance by repressing auxin signaling. Science.

[20] Zhang W., Gao S., Zhou X., Chellappan P., Chen Z., Zhou X., Zhang X., Fromuth N., Coutino G., Coffey M. (2011). Bacteria-responsive microRNAs regulate plant innate immunity by modulating plant hormone networks. Plant Mol. Biol..

[21] Li Y., Zhang Q., Zhang J., Wu L., Qi Y., Zhou J.M. (2010). Identification of microRNAs involved in pathogen-associated molecular pattern-triggered plant innate immunity. Plant Physiol..

[22] Xie Z., Johansen L.K., Gustafson A.M., Kasschau K.D., Lellis A.D., Zilberman D., Jacobsen S.E., Carrington J.C. (2004). Genetic and functional diversification of small RNA pathways in plants. PLoS Biol..

[23] Zhao M., Cai C., Zhai J., Lin F., Li L., Shreve J., Thimmapuram J., Hughes T.J., Meyers B.C., Ma J. (2015). Coordination of MicroRNAs, PhasiRNAs, and NB-LRR Genes in Response to a Plant Pathogen: Insights from Analyses of a Set of Soybean Rps Gene Near-Isogenic Lines. Plant Genome.

[24] Lan T., Yang X.Y., Chen J.W., Tian P., Shi L.N., Yu Y., Liu L., Gao L., Mo B.X., Chen X.M. (2022). Mechanism for the genomic and functional evolution of the MIR2118 family in the grass lineage. New Phytol..

[25] Borrelli G.M., Mazzucotelli E., Marone D., Crosatti C., Michelotti V., Valè G., Mastrangelo A.M. (2018). Regulation and Evolution of NLR Genes: A Close Interconnection for Plant Immunity. Int. J. Mol. Sci..

[26] Katiyar-Agarwal S., Morgan R., Dahlbeck D., Borsani O., Villegas A., Zhu J.K., Staskawicz B.J., Jin H.L. (2006). A pathogen-inducible endogenous siRNA in plant immunity. Proc. Natl. Acad. Sci. USA.

[27] Xie J., Tian J., Du Q., Chen J., Li Y., Yang X., Li B., Zhang D. (2016). Association genetics and transcriptome analysis reveal a gibberellin-responsive pathway involved in regulating photosynthesis. J. Exp. Bot..

[28] Persson S., Wei H., Milne J., Page G.P., Somerville C.R. (2005). Identification of genes required for cellulose synthesis by regression analysis of public microarray data sets. Proc. Natl. Acad. Sci. USA.

[29] Denancé N., Sánchez-Vallet A., Goffner D., Molina A. (2013). Disease resistance or growth: The role of plant hormones in balancing immune responses and fitness costs. Front. Plant Sci..

[30] Tuskan G.A., Difazio S., Jansson S., Bohlmann J., Grigoriev I., Hellsten U., Putnam N., Ralph S., Rombauts S., Salamov A. (2006). The genome of black cottonwood, Populus trichocarpa (Torr. & Gray). Science.

[31] Hacquard S., Joly D.L., Lin Y.C., Tisserant E., Feau N., Delaruelle C., Legué V., Kohler A., Tanguay P., Petre B. (2012). A Comprehensive Analysis of Genes Encoding Small Secreted Proteins Identifies Candidate Effectors in *Melampsora larici-populina* (Poplar Leaf Rust). Mol. Plant-Microbe Interact..

[32] Ma T., Wang J., Zhou G., Yue Z., Hu Q., Chen Y., Liu B., Qiu Q., Wang Z., Zhang J. (2013). Genomic insights into salt adaptation in a desert poplar. Nat. Commun..

[33] Du Q.Z., Wang B.W., Wei Z.Z., Zhang D.Q., Li B.L. (2012). Genetic Diversity and Population Structure of Chinese White Poplar (*Populus tomentosa*) Revealed by SSR Markers. J. Hered..

[34] Dodds P.N., Rathjen J.P. (2010). Plant immunity: Towards an integrated view of plant-pathogen interactions. Nat. Rev. Genet..

[35] Kim D., Pertea G., Trapnell C., Pimentel H., Kelley R., Salzberg S.L. (2013). TopHat2: Accurate alignment of transcriptomes in the presence of insertions, deletions and gene fusions. Genome Biol..

[36] Langmead B., Salzberg S.L. (2012). Fast gapped-read alignment with Bowtie 2. Nat. Methods.

[37] Ghosh S., Chan C.-K.K. (2016). Analysis of RNA-Seq Data Using TopHat and Cufflinks. Methods Mol. Biol..

[38] Johnson C., Kasprzewska A., Tennessen K., Fernandes J., Nan G.L., Walbot V., Sundaresan V., Vance V., Bowman L.H. (2012). Clusters and superclusters of phased small RNAs in the developing inflorescence of rice (vol 19, pg 1429, 2009). Genome Res..

[39] Sperschneider J., Gardiner D.M., Dodds P.N., Tini F., Covarelli L., Singh K.B., Manners J.M., Taylor J.M. (2016). EffectorP: Predicting fungal effector proteins from secretomes using machine learning. New Phytol..

[40] Dai X.B., Zhao P.X. (2011). psRNATarget: A plant small RNA target analysis server. Nucleic Acids Res..

[41] Xie J., Li Y., Liu X., Zhao Y., Li B., Ingvarsson P.K., Zhang D. (2019). Evolutionary Origins of Pseudogenes and Their Association with Regulatory Sequences in Plants. Plant Cell.

[42] Duplessis S., Hacquard S., Delaruelle C., Tisserant E., Frey P., Martin F., Kohler A. (2011). *Melampsora larici*-*populina* Transcript Profiling During Germination and Timecourse Infection of Poplar Leaves Reveals Dynamic Expression Patterns Associated with Virulence and Biotrophy. Mol. Plant-Microbe Interact..

[43] Weiberg A., Wang M., Lin F.M., Zhao H., Zhang Z., Kaloshian I., Huang H.D., Jin H. (2013). Fungal small RNAs suppress plant immunity by hijacking host RNA interference pathways. Science.

[44] Xie J., Yang X., Song Y., Du Q., Li Y., Chen J., Zhang D. (2017). Adaptive evolution and functional innovation of Populus-specific recently evolved microRNAs. New Phytol..

[45] Lu S., Li Q., Wei H., Chang M.J., Tunlaya-Anukit S., Kim H., Liu J., Song J., Sun Y.H., Yuan L. (2013). Ptr-miR397a is a negative regulator of laccase genes affecting lignin content in Populus trichocarpa. Proc. Natl. Acad. Sci. USA.

[46] Nova-Franco B., Íñiguez L.P., Valdés-López O., Alvarado-Affantranger X., Leija A., Fuentes S.I., Ramírez M., Paul S., Reyes J.L., Girard L. (2015). The micro-RNA72c-APETALA2-1 node as a key regulator of the common bean-Rhizobium etli nitrogen fixation symbiosis. Plant Physiol..

[47] Wang H., Wang H. (2015). The miR156/SPL Module, a Regulatory Hub and Versatile Toolbox, Gears up Crops for Enhanced Agronomic Traits. Mol. Plant.

[48] Slootweg E.J., Spiridon L.N., Roosien J., Butterbach P., Pomp R., Westerhof L., Wilbers R., Bakker E., Bakker J., Petrescu A.J. (2013). Structural determinants at the interface of the ARC2 and leucine-rich repeat domains control the activation of the plant immune receptors Rx1 and Gpa2. Plant Physiol..

[49] Lu C., Kulkarni K., Souret F.F., MuthuValliappan R., Tej S.S., Poethig R.S., Henderson I.R., Jacobsen S.E., Wang W., Green P.J. (2006). MicroRNAs and other small RNAs enriched in the Arabidopsis RNA-dependent RNA polymerase-2 mutant. Genome Res..

[50] Subramanian S., Fu Y., Sunkar R., Barbazuk W.B., Zhu J.K., Yu O. (2008). Novel and nodulation-regulated microRNAs in soybean roots. BMC Genom..

[51] Massoni J., Couvreur T.L., Sauquet H. (2015). Five major shifts of diversification through the long evolutionary history of Magnoliidae (angiosperms). BMC Evol. Biol..

[52] Zhao M., Meyers B.C., Cai C., Xu W., Ma J. (2015). Evolutionary patterns and coevolutionary consequences of MIRNA genes and microRNA targets triggered by multiple mechanisms of genomic duplications in soybean. Plant Cell.

[53] Le Q.H., Wright S., Yu Z., Bureau T. (2000). Transposon diversity in Arabidopsis thaliana. Proc. Natl. Acad. Sci. USA.

[54] Tomita M. (2010). Revolver and superior: Novel transposon-like gene families of the plant kingdom. Curr. Genom..

[55] Zilberman D., Cao X., Johansen L.K., Xie Z., Carrington J.C., Jacobsen S.E. (2004). Role of Arabidopsis ARGONAUTE4 in RNA-directed DNA methylation triggered by inverted repeats. Curr. Biol..

[56] Zhong X., Du J., Hale C.J., Gallego-Bartolome J., Feng S., Vashisht A.A., Chory J., Wohlschlegel J.A., Patel D.J., Jacobsen S.E. (2014). Molecular mechanism of action of plant DRM de novo DNA methyltransferases. Cell.

[57] Lisch D. (2009). Epigenetic regulation of transposable elements in plants. Annu. Rev. Plant Biol..

[58] Cho J. (2018). Transposon-Derived Non-coding RNAs and Their Function in Plants. Front. Plant Sci..

[59] Grandbastien M., Audeon C., Bonnivard E., Casacuberta J.M., Chalhoub B., Costa A.P.P., Le Q.H., Melayah D., Petit M., Poncet C. (2005). Stress activation and genomic impact of Tnt1 retrotransposons in Solanaceae. Cytogenet. Genome Res..

[60] Cavrak V.V., Lettner N., Jamge S., Kosarewicz A., Bayer L.M., Scheid O.M. (2014). How a Retrotransposon Exploits the Plant’s Heat Stress Response for Its Activation. PLoS Genet..

[61] Matsunaga W., Ohama N., Tanabe N., Masuta Y., Masuda S., Mitani N., Yamaguchi-Shinozaki K., Ma J.F., Kato A., Ito H. (2015). A small RNA mediated regulation of a stress-activated retrotransposon and the tissue specific transposition during the reproductive period in Arabidopsis. Front. Plant Sci..

[62] Ito H. (2013). Small RNAs and regulation of transposons in plants. Genes Genet. Syst..

[63] Ohtsu K., Smith M.B., Emrich S.J., Borsuk L.A., Zhou R., Chen T., Zhang X., Timmermans M.C., Beck J., Buckner B. (2007). Global gene expression analysis of the shoot apical meristem of maize (*Zea mays* L.). Plant J..

[64] McCue A.D., Nuthikattu S., Reeder S.H., Slotkin R.K. (2012). Gene expression and stress response mediated by the epigenetic regulation of a transposable element small RNA. PLoS Genet..

[65] Wei L., Gu L., Song X., Cui X., Lu Z., Zhou M., Wang L., Hu F., Zhai J., Meyers B.C. (2014). Dicer-like 3 produces transposable element-associated 24-nt siRNAs that control agricultural traits in rice. Proc. Natl. Acad. Sci. USA.

[66] Wang M., Weiberg A., Lin F.M., Thomma B.P., Huang H.D., Jin H. (2016). Bidirectional cross-kingdom RNAi and fungal uptake of external RNAs confer plant protection. Nat. Plants.

[67] Mackintosh C.A., Lewis J., Radmer L.E., Shin S., Heinen S.J., Smith L.A., Wyckoff M.N., Dill-Macky R., Evans C.K., Kravchenko S. (2007). Overexpression of defense response genes in transgenic wheat enhances resistance to Fusarium head blight. Plant Cell Rep..

[68] Weiberg A., Wang M., Bellinger M., Jin H. (2014). Small RNAs: A new paradigm in plant-microbe interactions. Annu. Rev. Phytopathol..

[69] Sin Y.W., Annavi G., Dugdale H.L., Newman C., Burke T., MacDonald D.W. (2014). Pathogen burden, co-infection and major histocompatibility complex variability in the European badger (Meles meles). Mol. Ecol..

[70] Zhai J., Jeong D.H., De Paoli E., Park S., Rosen B.D., Li Y., González A.J., Yan Z., Kitto S.L., Grusak M.A. (2011). MicroRNAs as master regulators of the plant NB-LRR defense gene family via the production of phased, trans-acting siRNAs. Genes Dev..

[71] Cai Q., Qiao L.L., Wang M., He B.Y., Lin F.M., Palmquist J., Huang S.N.D., Jin H.L. (2018). Plants send small RNAs in extracellular vesicles to fungal pathogen to silence virulence genes. Science.

[72] He B.Y., Wang H., Liu G.S., Chen A., Calvo A., Cai Q., Jin H.L. (2023). Fungal small RNAs ride in extracellular vesicles to enter plant cells through clathrin-mediated endocytosis. Nat. Commun..

[73] Buck A.H., Coakley G., Simbari F., McSorley H.J., Quintana J.F., Le Bihan T., Kumar S., Abreu-Goodger C., Lear M., Harcus Y. (2014). Exosomes secreted by nematode parasites transfer small RNAs to mammalian cells and modulate innate immunity. Nat. Commun..

[74] Hinas A., Wright A.J., Hunter C.P. (2012). SID-5 Is an Endosome-Associated Protein Required for Efficient Systemic RNAi in *C. elegans*. Curr. Biol..

[75] Cunnac S., Chakravarthy S., Kvitko B.H., Russell A.B., Martin G.B., Collmer A. (2011). Genetic disassembly and combinatorial reassembly identify a minimal functional repertoire of type III effectors in Pseudomonas syringae. Proc. Natl. Acad. Sci. USA.

[76] Ninio S., Celli J., Roy C.R. (2009). A Legionella pneumophila effector protein encoded in a region of genomic plasticity binds to Dot/Icm-modified vacuoles. PLoS Pathog..

[77] Meile L., Croll D., Brunner P.C., Plissonneau C., Hartmann F.E., McDonald B.A., Sánchez-Vallet A. (2018). A fungal avirulence factor encoded in a highly plastic genomic region triggers partial resistance to septoria tritici blotch. New Phytol..

[78] Borges F., Martienssen R.A. (2015). The expanding world of small RNAs in plants. Nat. Rev. Mol. Cell Biol..

[79] Stitzer M.C., Anderson S.N., Springer N.M., Ross-Ibarra J.J.P.G. (2021). The genomic ecosystem of transposable elements in maize. PLoS Genet..

[80] Jones J.D.G., Dangl J.L. (2006). The plant immune system. Nature.

[81] Innes R.W., Ameline-Torregrosa C., Ashfield T., Cannon E., Cannon S.B., Chacko B., Chen N.W.G., Couloux A., Dalwani A., Denny R. (2008). Differential Accumulation of Retroelements and Diversification of NB-LRR Disease Resistance Genes in Duplicated Regions following Polyploidy in the Ancestor of Soybean. Plant Physiol..

[82] Fei Q.L., Xia R., Meyers B.C. (2013). Phased, Secondary, Small Interfering RNAs in Posttranscriptional Regulatory Networks. Plant Cell.

[83] Karasov T.L., Horton M.W., Bergelson J. (2014). Genomic variability as a driver of plant-pathogen coevolution?. Curr. Opin. Plant Biol..

[84] Li Y.H., Zhou G., Ma J., Jiang W., Jin L.G., Zhang Z., Guo Y., Zhang J., Sui Y., Zheng L. (2014). De novo assembly of soybean wild relatives for pan-genome analysis of diversity and agronomic traits. Nat. Biotechnol..

[85] Rhoades M.W., Reinhart B.J., Lim L.P., Burge C.B., Bartel B., Bartel D.P. (2002). Prediction of plant microRNA targets. Cell.

[86] Chen K., Rajewsky N. (2007). The evolution of gene regulation by transcription factors and microRNAs. Nat. Rev. Genet..

[87] Rodgers-Melnick E., Mane S.P., Dharmawardhana P., Slavov G.T., Crasta O.R., Strauss S.H., Brunner A.M., Difazio S.P. (2012). Contrasting patterns of evolution following whole genome versus tandem duplication events in Populus. Genome Res..

