# Supplementary material for: Transposon-Associated Small RNAs Involved in Plant Defense in Poplar
Source: Plants (Basel). 2025 Apr 21;14(8):1265. doi: 10.3390/plants14081265 (PMC12030527; doi:10.3390/plants14081265)
Supplement: Supplementary file 1 [file plants-14-01265-s001.zip › Table S1.pdf]

Table S1. Small RNA sequencing reads produced for the three samples.

Samples	Total Reads	Mapped reads	Mapping rate
T01_rep1	15330278	5132868	0.335
T01_rep2	14854207	5856875	0.394
T02_rep1	12899207	5316866	0.412
T02_rep2	13598494	5546789	0.408
T03_rep1	11982109	4820944	0.402
T03_rep2	12648897	5564897	0.44
